# Glances at the Hospitals

**Published:** 1904-09-03

**Authors:** 


### GLANCES AT THE HOSPITALS.

### NEWCASTLE-ON-TYNE HOSPITAL FOR SICK CHILDREN.

The income was £3,625, and the expenditure was £3,424. The workpeople's contributions amounted to more than £200. On January 1st the hospital contained 65 children, and 742 were admitted during the year, making a total of 807 under treatment. Of these, 367 were discharged cured, 171 relieved, 89 not relieved, 51 died, and 64 remained in the wards. The death-rate in the surgical wards was 3·3 per cent., and in the medical ward 11·2 per cent. The average daily number resident was 67, and the average stay in the hospital was 27 days. The medical and surgical tables are well arranged, and are divided into columns showing the number of cases, the numbers cured, relieved, not relieved, died, and remaining under treatment. That common omission from hospital statistics, an anæsthetic table, has to be mentioned.

